# Protective Effect of Melatonin Against Polymicrobial Sepsis Is Mediated by the Anti-bacterial Effect of Neutrophils

**DOI:** 10.3389/fimmu.2019.01371

**Published:** 2019-06-19

**Authors:** Li Xu, Wei Zhang, Minseok Kwak, LiJun Zhang, Peter C. W. Lee, Jun-O Jin

**Affiliations:** ^1^Shanghai Public Health Clinical Center, Institutes of Biomedical Sciences, Shanghai Medical College, Fudan University, Shanghai, China; ^2^Department of Chemistry, Pukyong National University, Busan, South Korea; ^3^Department of Biomedical Sciences, ASAN Medical Center, University of Ulsan College of Medicine, Seoul, South Korea; ^4^Department of Medical Biotechnology, Yeungnam University, Gyeongsan, South Korea

**Keywords:** melatonin, anti-bacterial effect, neutrophil extracellular traps, polymicrobial infection, sepsis

## Abstract

Sepsis is an infection- or toxin-mediated systemic inflammatory syndrome. Previous studies have shown that melatonin, the primary hormone produced by the pineal gland, attenuates the effect of polymicrobial infection-mediated septic shock in animals. However, the mechanism of the anti-septic effect of melatonin during polymicrobial infection has not been well-studied. In this study, we investigated how melatonin protects mice from polymicrobial sepsis. Melatonin treatment inhibited peripheral tissue inflammation and tissue damage in a cecal ligation puncture (CLP)-induced polymicrobial sepsis model, consequently reducing the mortality of the mice. We found that macrophages and neutrophils expressed melatonin receptors. Upon depletion of neutrophils, melatonin-induced protection against polymicrobial infection failed in the mice, but melatonin treatment in macrophage-depleted mice attenuated the mice mortality resulting from polymicrobial sepsis. Moreover, melatonin treatment promoted the development of the neutrophil extracellular trap (NET), which contributed to anti-bacterial activity during polymicrobial infection, whereas the phagocytic activities of neutrophils were inhibited by melatonin. The data from this study support previously unexplained antiseptic effects of melatonin during a polymicrobial infection and could be potentially useful for human patients with sepsis.

## Introduction

Sepsis is a systemic inflammatory response syndrome caused by an infection, which can lead to multiple organ dysfunction and mortality. Sepsis affects millions of people worldwide annually and has a 30–50% mortality rate (1). Sepsis as an acute response to a pathogen or its toxin in the early stage promotes the elevation of pro-inflammatory cytokine levels, also known as cytokine storm, leading to high fever, tachycardia, and tachypnea (2). Overexpression of pro-inflammatory cytokines is required for the clearance of the invading pathogens, although they also contribute to tissue inflammation and multi-organ dysfunction (2–4). To prevent a septic cytokine storm, anti-inflammatory reagents have been used in patients (5, 6). However, treatment with anti-inflammatory reagents typically fails to recover the sepsis, as they also inhibit the clearance of the invading pathogen (4). Therefore, treatment with antibiotics to effectively kill pathogens during the early stages may be the best treatment method for sepsis (7, 8).

Activation of immune cells contributes to clearance of bacterial infection. Neutrophils are the most abundant leukocytes in the peripheral blood, and they are activated the earliest stages of inflammatory reaction against bacterial infection (9, 10). Neutrophils are a critical participant during the initial immune effort to eliminate invading pathogens. Neutrophils effectively kill bacteria through phagocytosis (11) and neutrophil extracellular traps (NETs) (12–14). NETs represent an innate defense mechanism against invading pathogens, in which concentrated anti-microbial molecules are mobilized to the infected area to kill and prevent the spread of pathogens (12, 13). During NET formation, the function of neutrophils against the bacteria is disrupted (9, 12) as the neutrophils release granule proteins and chromatin to form the extracellular fibril matrix (13, 14).

In addition to modulating the dark-light cycle, melatonin, the primary chronobiotic hormone, plays important roles in the neuroendocrine-immunological network. The therapeutic effects of melatonin have been reported in several disorders, such as certain tumors, cardiovascular diseases, and psychiatric disorders (15–17). Meanwhile, melatonin also participates in the regulation of innate immunity (18). Moreover, it has been reported that melatonin has pro-apoptotic effects in the human myeloid cell line HL-60 (19). Melatonin has also been shown to be beneficial in reversing the effect of septic shock (20–23). Melatonin induces an anti-inflammatory response and an anti-oxidant effect, which decreases the production of pro-inflammatory cytokines and the release of nitric oxide (NO) (20, 21, 24). The anti-inflammatory properties of melatonin offer therapeutic protection in animal sepsis models, such as lipopolysaccharide (LPS)-induced septic shock in mice (22, 25, 26), peritonitis-induced sepsis in rats (20, 21), and cecal ligation puncture (CLP)-induced polymicrobial sepsis model (27–29). Moreover, melatonin also shows anti-apoptotic effects by inhibiting inflammasome and mitochondrial dysfunction in polymicrobial sepsis models (30, 31). Melatonin acts through different molecular pathways, the best characterized being the activation of two types of membrane specific receptors: MT1 and MT2. They are expressed in a wide variety of organs and immune cells, which regulate cytokine expression and signaling pathway activation (32). In addition, melatonin receptors play an important role in promoting bacterial phagocytosis (32). Although melatonin has been shown to protect from sepsis, this effect may not be mediated through its anti-inflammatory function, since inhibition of inflammation has limited effects on protecting mice against polymicrobial infection-mediated sepsis (4–6). Effective therapeutic strategies against polymicrobial sepsis, should result in clearance of the invading pathogens (7, 8, 33, 34). How melatonin contributes to the clearance of the invading pathogens in polymicrobial-infected septic models is not fully understood (32). In this study, we found that neutrophils and macrophages express melatonin receptors. We hypothesized that they may contribute to the clearance of the invading bacteria upon melatonin treatment and aimed to examine this hypothesis.

## Materials and Methods

### Mice

C57BL/6 mice (6–10 weeks old) were obtained from the Shanghai Public Health Clinical Center (SPHCC) and housed under pathogen-free conditions. The mice were maintained at a temperature of 20–22°C and humidity of 50–60%, under a cycle of 12 h light and 12 h dark. The mice had free access to water and standard rodent chow. All the experiments were carried out under the guidelines of the Institutional Animal Care and Use Committee at the SPHCC. The mouse method (2018-A050-01) was approved by the committee on the Ethics of Animal Experiments of the SPHCC. For sacrificing the mice, CO_2_ inhalation euthanasia was used.

### Chemicals and Cytokines

Melatonin and luzindole were obtained from Sigma-Aldrich (St. Louis, MO, USA). Melatonin was dissolved to a stocking concentration of 10 mg/mL sterile water. Luzindole in a concentration of 1 mg/ml was dissolved in PBS including 5% DMSO. Annexin V & PI staining kit was purchased from BioLegend (San Diego, CA, USA). For flow cytometry analysis, isotype control antibodies (Abs), anti-Ly-6G Abs (1A8), and anti-F4/80 Abs (BM8) were obtained from BioLegend (San Diego, CA, USA).Anti-histone H3 Abs (ab5103) were purchased from Abcam (Cambridge, UK). Anti-GAPDH was obtained from transgen (Beijing, China).

### Flow Cytometry Analysis

The cells were incubated with isotype control Abs (unlabeled) and Fc-block for 20 min (BioLegend, San Diego, CA, USA). Next, the cells were incubated with fluorescence-conjugated Abs on ice for 20 min and, washed with PBS and analyzed by flow cytometry (Becton Dickinson, Franklin Lakes, New Jersey, USA). Dead cells and debris were excluded by 4′,6-diamidino-2-phenylindole (DAPI, 5 μM) staining (Sigma-Aldrich, St. Louis, MO, USA) and gating of forward- and side-scatter. The antibodies used in flow cytometry were titrated and their final concentrations for staining were defined.

### CLP Surgery

CLP surgery was performed as described elsewhere (35). Briefly, after the mice were anesthetized intraperitoneally (*i.p*.) with an injection of 100 mg/kg ketamine and 10 mg/kg xylazine, an incision was made in the abdomen. After the cecum was exposed, it was ligated below the ileocecal junction, and a single puncture in the cecum was made using an 18-gauge needle. Control mice (sham) were subjected to the same procedures, except for the cecal puncture. All mice underwent the CLP surgery at similar times of the day (09:00 am) to avoid any diurnal variation effect.

### Histology and Immunofluorescence Staining

Following a previous study (36), the lung and liver of the mice were fixed in 4% paraformaldehyde and incubated in ethanol for 24 h at 4°C. The tissues were then embedded in paraffin, and sections of 5 μm thickness were prepared from different regions of the tissue. The tissue samples were stained with hematoxylin and eosin (H&E), and the presence of leukocytes was examined under a microscope. For the NETs formation assay, peritoneal neutrophils were isolated 6 h after CLP. Cells underwent cytospin, were fixed with 4% paraformaldehyde and stained with SYTOX Green (Invitrogen, Carlsbad, CA, USA, 5 μM) for 15 min. Or, cells were incubated overnight with primary biotin conjugated anti-Ly-6G Abs (1A8) and anti-histone H3 Abs (citrulline R2 + R8 + R17, diluted at 1:250) at 4°C, followed by incubation with Alexa 594-conjugated streptavidin (Invitrogen, Carlsbad, CA, USA, diluted at 1:1000) and Alexa488-conjugated anti-rabbit Abs (Invitrogen, Carlsbad, CA, USA, diluted at 1:500) for 2 h. The stained samples were examined using a Leica laser scanning confocal microscope (Leica Microsystems, Wetzlar, Germany). Images were taken using the Leica confocal software (Leica Microsystems).

### Bacterial Counts

Tissues were harvested and homogenized in 1 mL of PBS. Then, 50 μL volumes of the homogenates were cultured overnight on blood agar plates. The peritoneal fluid and BALF were also collected from mice after CLP surgery and cultured overnight on blood agar plates.

### Detection of H3-Cit Levels From Peritoneal Fluid

Six hours after the CLP surgery, the peritoneal fluid was harvested, and the secreted proteins in the supernatants were collected and concentrated using speed-vac. The protein concentration was determined by the Bicinchoninic Acid (BCA) method. Equal amounts of proteins were separated by SDS-PAGE and transferred to a polyvinylidene difluoride membrane (Millipore, Burlington, MS, USA). Blots were blocked with 5% milk-PBST for 1 h and probed with primary antibody against H3-cit (citrulline R2 + R8 + R17, from Abcam, Cambridge, UK, diluted at 1:1000). After incubation with secondary antibody and detection by Enhanced Chemiluminescence Plus (GE Healthcare), protein bands were scanned using an LI-COR Odyssey Fc imaging system.

### Immune Cell Isolation

T cells, B cells, NK cells, and DCs were isolated from the spleen using a Pan T Cell Isolation Kit II, a Pan B Cell Isolation Kit II, a Macrophage Isolation Kit, and a Pan DC Isolation Kit, respectively (Miltenyi Biotec, Bergisch Gladbach, Germany). Neutrophils were isolated from bone marrow cells using the Percoll gradient (Sigma-Aldrich, St. Louis, MO, USA), as described elsewhere (37, 38). Briefly, bone marrow cells were extracted from mouse femurs and tibias. After removal of red blood cells by the ACK buffer (Beyotime, Shanghai, China), cells were carefully loaded on a 52, 69, 78% percoll (GE Healthcare) gradient, and centrifuged at 1500 g for 30 min without breaking. Cells were isolated on the 69/78% interface layer. In some experiments, peritoneal neutrophils were isolated after CLP surgery. Pasteur pipettes were filled with cold PBS and inserted through the peritoneal wall to inject PBS into each mouse. After aspiration of the fluid from the peritoneum, the peritoneal fluid was slowly withdrawn. The cell suspension was then centrifuged at 1700 rpm for 5 min and washed with PBS.

### Bacterial Phagocytosis

*Escherichia Coli* (*E. Coli*) and *Staphylococcus Aureus* (*S. Aureus*) were labeled with cell-tracking fluorescent dyes PKH26 (red) and PKH67 (green) (Sigma-Aldrich, St. Louis, MO, USA), respectively. Neutrophils and macrophages (0.2 × 10^6^) were co-cultured with a mixture of *S. aureus* and *E. coli* (2 × 10^6^ total cell number) for 1 h. Phagocytic bacterial cells were defined as PKH67^+^ or PKH26^+^ cells in Ly-6G^+^ neutrophils and F4/80^+^ macrophages, by flow cytometry.

### Polymerase Chain Reaction (PCR)

cDNA were synthesized from the total RNA using M-MLV reverse transcriptase and oligonucleotides (dT) (Promega, Madison, Wisconsin, US). The cDNA was amplified in a DNA thermal cycler for 40 cycles using the PCR program (95°C for 1 min, 55°C for 1 min, and 72°C for 30 s). For real-time PCR, the cDNA was subjected to real-time PCR amplification (Qiagen, Hilden, Germany) for 40 cycles with an annealing and extension temperature of 60°C, on a Light Cycler 480 Real-Time PCR System (Roche, Basel, Switzerland).

### Neutrophil and Macrophage Depletion

The mice were injected *i.p*. with 500 μg anti-Ly-6G Abs (1A8 from BioXCell, West Lebanon, NH, USA) 24 and 2 h before CLP surgery; an injection of anti-Ly-6G Abs was repeated every 24 h. Neutrophil depletion was confirmed by flow cytometry, and the depletion of the CD11b^+^Ly-6G^+^ cells was >99%. For the depletion of macrophages, the mice received an *i.p*. injection of clodronate liposomes 24 and 2 h before CLP surgery. The injection of clodronate liposomes was repeated every 24 h. Macrophage depletion was assessed by flow cytometry, which showed that >99% of the macrophages were depleted.

### Elastase Assay

The EnzChek elastase assay kit (Molecular Probes, Eugene, OR, USA) was used according to the manufacturer-provided protocol. Fluorescence was detected using an X4 2030 Multilabel Reader fluorescence reader (Perkin Elmer, Waltham, MS, USA).

### MPO Assay

Serum and cultured medium were analyzed for MPO by ELISA following the manufacturer's protocol (Abcam, Cambridge, UK). Briefly, the serum or cultured medium was loaded to pre-coated wells. Reaction mixture was then added to the wells for 2 h followed by treatment with stop buffer. Ten minutes after, TMB reagent was added and the plate was analyzed at 450 nm.

### Statistical Analysis

All the data are presented as mean ± standard error of the mean (SEM). One-way ANOVA was used for analysis (followed by Tukey's multiple comparison test in Graph Pad Prism 4). The *p*-values <0.05 were considered statistically significant.

## Results

### Melatonin Prevented Mice Mortality Resulting From Cecal Ligation Puncture (CLP)-Induced Sepsis

**Figure 1:**
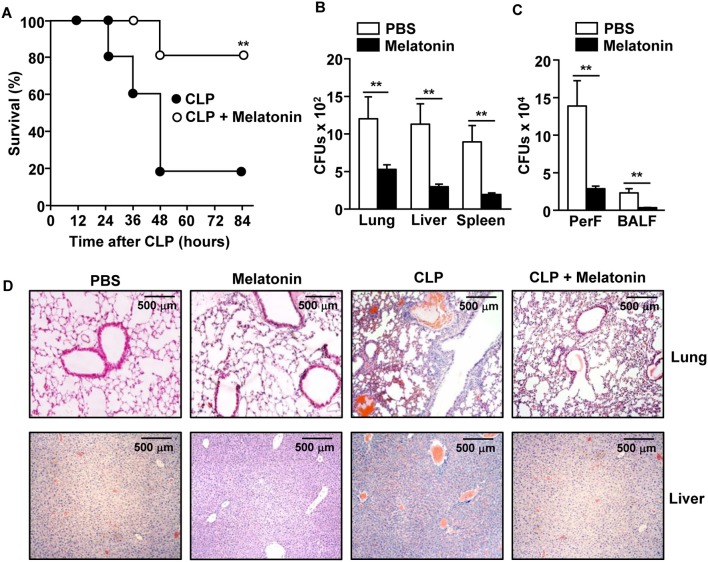
Melatonin attenuated the CLP-induced mortality in mice. C57BL/6 mice were injected with 50 mg/kg melatonin for 30 min before they underwent CLP surgery. Thirty minutes after the surgery, the mice received the same concentration of melatonin again. **(A)** The survival rate of the mice was monitored. **p < 0.01, based on ANOVA. Sample size, *n* = 10 per group. **(B)** Bacterial colony forming units (CFUs) were measured for homogenates of the lung, liver, and spleen, 24 h after the CLP surgery. **(C)** The CFUs in the peritoneal fluid (PerF) and bronchoalveolar lavage fluid (BALF). **(D)** Hematoxylin and eosin (H&E) staining of the lung and liver 24 h after CLP surgery. Data represent the average of six independent samples (two mice per experiment, for a total of three experiments). **p < 0.01.

To determine the anti-septic effect of melatonin, C57BL/6 mice were treated with melatonin before and after the CLP surgery. Consistent with previous studies (21, 27, 39, 40), the mortality of mice from CLP-induced sepsis was effectively reduced in mice treated with melatonin (Figure 1A). The CLP surgery induced an increase in the number of bacterial colony forming units (CFUs) in the lung, liver, and spleen tissues, as well as in the peritoneal and bronchoalveolar lavage fluids (BALF), while melatonin treatment dramatically reduced the bacterial CFUs in these tissues and fluids (Figures 1B,C). Moreover, melatonin treatment substantially inhibited the CLP-induced infiltration of leukocytes in the lungs and liver (Figure 1D).

Next, we examined peripheral tissue failure and found that CLP surgery promoted an increase in the lung wet/dry ratio, indicating the development of pulmonary edema, while melatonin treatment inhibited the increase in the lung wet/dry ratio (Supplementary Figure 1A). The increased levels of plasma aspartate aminotransferase (AST), a marker of liver damage, in CLP surgery mice, were also suppressed by melatonin (Supplementary Figure 1B). Moreover, melatonin treatment suppressed the CLP-induced liver and lung cell death, as indicated by the reduced TUNEL-positive cells (Supplementary Figure 2A). Melatonin also inhibited the apoptotic cell death in the spleen 24 h after CLP surgery (Supplementary Figure 2B). Furthermore, the elevated levels of pro-inflammatory cytokines resulting from CLP surgery were also substantially decreased upon melatonin treatment (Supplementary Figure 3). Thus, these data indicated that melatonin attenuated the CLP-induced tissue damage, bacterial colonization, inflammation, and mortality in the mice.

### Levels of Melatonin Receptor 2 in Neutrophils Were Upregulated During Bacterial Infection

**Figure 2:**
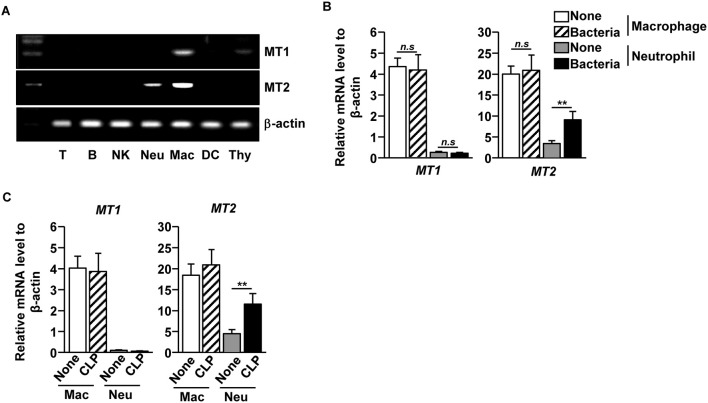
Elevation of melatonin receptor 2 (MT2) level in neutrophils upon bacterial infection. **(A)** T cells (T), B cells **(B)**, Consistent with the flow cytometry NK cells (NK), macrophages (Mac), and dendritic cells (DC) were isolated from the spleen, and the neutrophils (Neu) were purified from the bone marrow. The mRNA expression levels of melatonin receptors 1 and 2 (MT1 and MT2) were then measured in the isolated cells. Thymus (Thy) was used as a control. **(B)** Isolated macrophages and neutrophils were co-cultured with mixture of *S. aureus* and *E. coli* for 1 h, and the mRNA levels of MT1 and MT2 were measured using real-time qPCR. *n* = 6 per group, **p < 0.01. **(C)** MT1 and MT2 mRNA levels were measured in isolated macrophages and neutrophils 12 h after the CLP surgery in mice. Data are the average of six independent samples for each group. **p < 0.01.

As melatonin prevented bacterial growth and inflammation in the tissues and fluids of CLP-induced septic mice, we next examined which type of immune cells responded to melatonin during CLP-induced sepsis in mice. We first measured the mRNA levels of melatonin receptors 1 and 2 (MT1 and MT2) in the immune cells and found that the macrophages expressed both MT1 and MT2, while the neutrophils expressed MT2, in the naive mice. Other immune cells, including T cells, B cells, natural killer (NK) cells, and dendritic cells (DCs), did not express either MT1 or MT2 (Figure 2A). To measure how the receptor expression affects bacterial infection, isolated macrophages and neutrophils were infected with a mixture of *E. coli* and *S. aureus*. The bacterial infection significantly upregulated the MT2 mRNA levels in the neutrophils, whereas the levels of MT1 and MT2 mRNA in the macrophages did not change (Figure 2B). Consistent with the *in vitro* assay, MT2 levels in neutrophils were elevated after the CLP surgery, compared with control mice (Figure 2C). These data indicated that melatonin may act on neutrophils following bacterial infection.

### Melatonin Enhanced the Bactericidal Effects of Neutrophils

**Figure 3:**
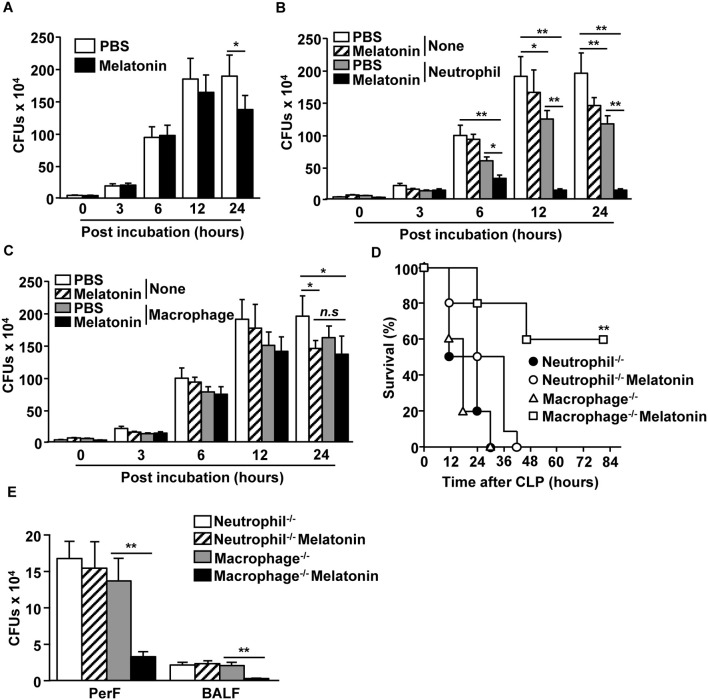
Neutrophils were required for melatonin-induced protection against polymicrobial-infected sepsis. **(A)** Mixture of *E. coli* and *S. aureus* were incubated with 100 μg/ml of melatonin. Anti-bacterial effect of melatonin was shown. (B) Neutrophils were isolated from the bone marrow, and the neutrophils (0.2 × 10^6^) were co-cultured with a mixture of *E. coli* (1 × 10^6^) and *S. aureus* (1 × 10^6^). Numbers of CFUs were measured in the PBS- and 100 μg/ml of melatonin-treated neutrophils. **(C)** Isolated macrophages from spleen were assessed for their anti-bacterial effects upon melatonin (100 μg/ml) treatment. All data are the average of six independent samples. *p < 0.05, **p < 0.01. **(D)** Survival rates were monitored after melatonin treatment in neutrophil- or macrophage-depleted mice after CLP surgery. **p < 0.01, based on ANOVA. Sample size, *n* = 10 per group. **(E)** The numbers of CFUs in the peritoneal fluid (PerF) and bronchoalveolar lavage fluid (BALF) were measured 24 h after the melatonin treatment and CLP surgery. **p < 0.01 from six independent samples.

We next examined whether neutrophils and macrophages contribute to the clearance of bacteria upon melatonin treatment. As melatonin has inherent antibiotic effects as well (41), we first examined the effect of melatonin treatment on a mixture of *E. coli* (DE 3) and *S. aureus* (NCTC 8532), and found that the bacterial CFUs were considerably decreased after 24 h of melatonin treatment (Figure 3A). We also examined whether melatonin treatment in neutrophils promotes the clearance of bacteria and found that melatonin treatment in neutrophils significantly reduced the number of bacterial CFUs compared with the melatonin-only treatment (Figure 3B). However, melatonin did not induce antibiotic effects in macrophages, as the bacterial CFU levels in this case were not different compared with melatonin-treated control (Figure 3C). In addition, melatonin treatment did not prevent the CLP-surgery-induced mortality in neutrophil-depleted mice, while the macrophage-depleted mice survived after CLP surgery upon melatonin treatment (Figure 3D). Moreover, the number of bacterial CFUs in peritoneal fluid and BALF were not decreased by melatonin treatment in neutrophil-depleted CLP mice, whereas melatonin treatment in macrophage-depleted CLP mice was able to inhibit bacterial CFUs (Figure 3E) Thus, these data suggested that melatonin-induced anti-bacterial effects are mediated by neutrophils.

### Melatonin Did Not Enhance the Phagocytic Activities of Neutrophils

**Figure 4:**
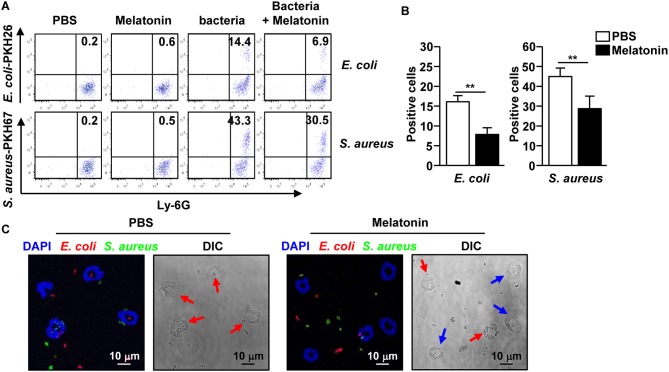
Melatonin suppressed the phagocytic activity in neutrophils. **(A)** Phagocytosis of PKH26-labeled *E. coli* and PKH67-labeled *S. aureus* by neutrophils was analyzed by flow cytometry. **(B)** The mean number of *E. coli* (left panel) and *S. aureus* (right panel) phagocytosed by the neutrophils are shown. **(C)** Isolated neutrophils were co-cultured with PKH26-labeled *E. coli* (red color) and PKH67-labeled *S. aureus* (green color) for 1 h, and the cells were spun down on a glass slide using a cytospin. Phagocytic neutrophils were observed used a confocal microscope. Red arrows indicate the phagocytic neutrophils and blue arrows indicate non-phagocytic neutrophils. Data represent the average of six independent samples for each group (three samples per experiment, total of two independent experiments), **p < 0.01.

As neutrophils contribute to bacterial clearance in mice via phagocytosis, we first evaluated the effect of melatonin on the phagocytic activity of neutrophils, and compared it with the effect on macrophages. The neutrophils and macrophages were first treated with melatonin for 1 h, following which, the cells were co-cultured with *E. coli* and *S. aureus* for an additional hour. The non-treated neutrophils effectively phagocytosed the *E. coli* and *S. aureus*, whereas the neutrophils treated with melatonin significantly decreased the phagocytosis of the bacteria (Figures 4A,B). The phagocytic activity of the macrophages was not affected by the melatonin treatment (Supplementary Figure 4). Consistent with the flow cytometry analysis, the confocal microscopy results showed that the melatonin treatment inhibited the phagocytosis of bacteria in the neutrophils (Figure 4C). Thus, these data indicated that the melatonin-induced anti-bacteria effects are not mediated by the phagocytic activity of neutrophils.

### Melatonin-Induced Neutrophil Extracellular Trap (NET) Formation

Our data showed that melatonin-treated neutrophils exhibited a bactericidal effect without inducing phagocytosis, which prompted us to examine the effect of melatonin on the induction of NET formation, as NET inhibits the phagocytic activity of neutrophils (13, 42). Isolated neutrophils were treated with melatonin, infected with a mixture of *E. coli* and *S. aureus* for 6 h, and the cells were stained with SYTOX Green, a non-permeable dye that stains nucleic acids, to determine NET formation. Bacterial infection led to a slight increase in the NET formation by neutrophils, whereas NET formation was markedly increased when the neutrophils were treated with melatonin and then infected with bacteria (Supplementary Figure 5A). Moreover, NET formation was dose dependently increased by melatonin (Supplementary Figure 5D). In addition, the active elastase and myeloperoxidase (MPO) levels were also significantly increased compared with other controls (Supplementary Figures 5B,C).

**Figure 5:**
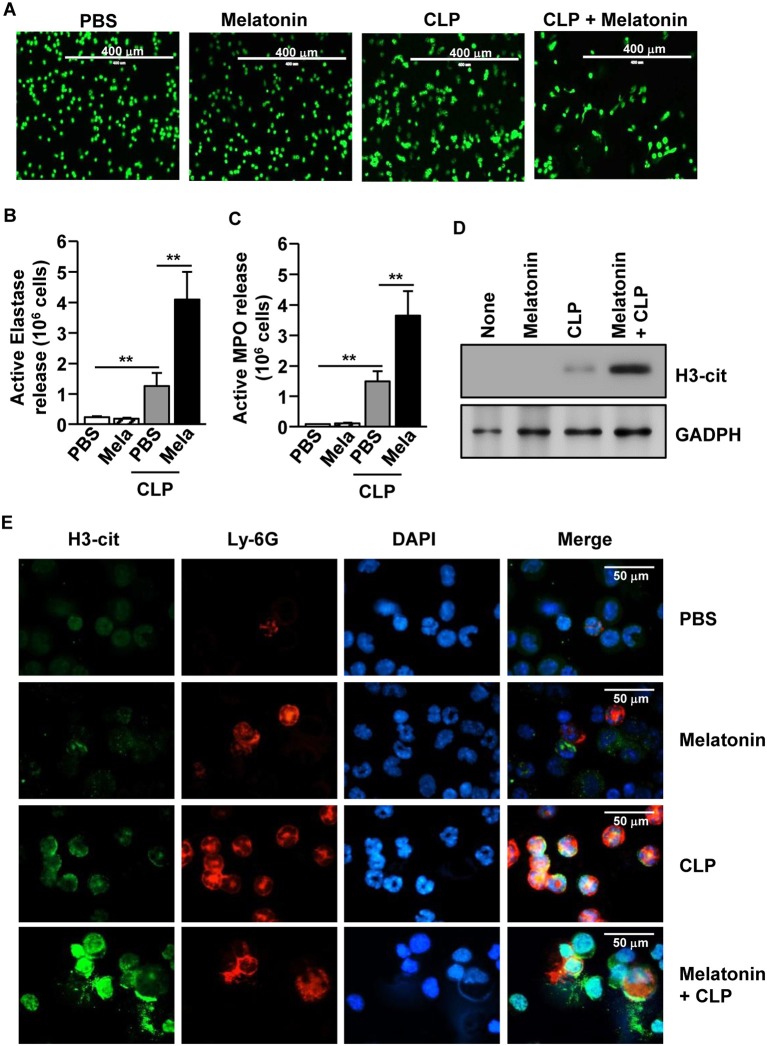
Melatonin-induced neutrophil extracellular traps (NETs) in polymicrobial infection. C57BL/6 Mice were treated with 50 mg/kg melatonin and then subjected to CLP surgery. The mice were further injected with 50 mg/kg melatonin 30 min after the CLP surgery. **(A)** Peritoneal neutrophils were harvested and stained with SYTOX Green, 6 h after the CLP surgery. **(B)** The active elastase and **(C)** myeloperoxidase (MPO) levels were measured in the peritoneal fluid. **(D)** Secreted protein levels of cit-H3 were measured in the supernatant of the peritoneal fluid. **(E)** The peritoneal cells were analyzed by immunofluorescence staining with Ly-6G, H3-cit, and DAPI. All data represent the average of six independent samples (two mice per experiment, for a total of three experiments), *p < 0.01.

We also examined whether melatonin treatment could induce NETs in CLP-induced polymicrobial-infected mice. The CLP-induced polymicrobial infection caused a considerable increase in the NETs in the peritoneal neutrophils. Moreover, the levels were substantially increased after melatonin treatment, as indicated by the changes in the nuclear morphology (Figure 5A). Consistent with the *in vitro* data, after the CLP surgery, the active elastase and MPO levels were significantly increased in the peritoneal fluid (Figures 5B,C). In addition, the release levels of citrullinated histone H3 (H3-cit), a marker of NETs, were markedly increased in the peritoneum of polymicrobial-infected mice upon melatonin treatment, compared with the controls (Figures 5D,E). Therefore, these data suggested that melatonin promoted the development of NETs in CLP-induced polymicrobial-infected mice.

As melatonin promoted NET formation in polymicrobial-infected mice, we further examined whether the NETs were essential for protection against polymicrobial infection. The mice were pretreated *i.p*. with 5 mg/kg luzindole, an inhibitor of melatonin, for 1 h, after which, the mice received melatonin. After 30 min, the mice underwent CLP surgery and were treated with 50 mg/kg melatonin. Melatonin-induced inhibition of lung and liver damage, and the anti-apoptotic effect in splenocytes was markedly reduced by luzindole (Supplementary Figures 6A,B). Furthermore, the melatonin-inhibited infiltration of leukocytes in the lungs and liver (Supplementary Figure 6C), as well as the pro-inflammatory cytokine levels (Supplementary Figure 6D), were also diminished by luzindole in the CLP surgery mice. In addition, the melatonin-inhibited bacterial growth in the peritoneal fluid and BALF were abrogated by luzindole in the CLP-induced polymicrobial-infected mice (Supplementary Figure 6E). Together, these data suggested that luzindole suppressed the melatonin-induced inhibition of tissue damage, inflammation, and antibacterial effects in CLP-induced polymicrobial-infected mice.

We further examined whether luzindole-induced suppression of melatonin effects was mediated by the inhibition of NETs. Treatment with luzindole substantially inhibited the melatonin-induced NETs in polymicrobial-infected mice, as shown by the inhibition of morphological changes of neutrophils in Supplementary Figure 7A, and decreased release levels of H3-cit in Supplementary Figures 7B,C. In addition, the elevated release levels of active elastase and MPO in response to melatonin were also dramatically decreased by luzindole in the peritoneal fluid of CLP surgery mice (Supplementary Figures 7D,E). Thus, these data suggested that luzindole inhibited the melatonin-induced NETs, which consequently suppressed the protective effect of melatonin in CLP-mediated polymicrobial sepsis in mice.

## Discussion

Melatonin has been shown to have beneficial effects in the treatment of sepsis and inflammation (22, 23, 30, 43, 44). Treatment with melatonin contributed to the inhibition of nuclear factor kappa-light-chain-enhancer of activated B cells (NF-κB) and NACHT, LRR, and PYD domain-containing protein 3 (NLRP3) pathway, which are essential components of inflammasomes (45). Moreover, melatonin regulates apoptosis and autophagy in tissue, further contributing to the protection against sepsis (22). The apoptosis, inflammation, and tissue damage in CLP mice are mediated by polymicrobial infection (46). Although melatonin protects mice from CLP-induced polymicrobial infection by inhibition of apoptosis, inflammation, and tissue damage, it is still not clear how melatonin treatment contributes to the clearance of bacterial pathogens. In this study, we found that melatonin promoted NETs, which induced effective killing of bacteria. NETs also have an antiseptic effect through the clearance of bacteria (13). Thus, these data suggested that the protective effect of melatonin against CLP-induced sepsis is preferentially initiated by the enhanced formation of NETs.

MT1 and MT2 help in the protection against CLP-induced sepsis in mice, while the inhibition of MT1 and MT2 suppresses the melatonin-induced protective effects (47), although it has not been reported what types of cells contribute to these effects. Our findings support previous results, as we found that neutrophils expressed MT2, and the inhibition of MT2 suppressed the melatonin-induced protective effect in the CLP-induced polymicrobial-infected mice. Moreover, the inhibition of MT1 and MT2 by luzindole prevented NET formation in polymicrobial-infected mice, and consequently, the invading bacteria were not cleared in the mice. We also found that the macrophages expressed both MT1 and MT2. It has been shown that melatonin treatment inhibits the LPS-induced production of pro-inflammatory cytokines and NO production in macrophages (18). We also showed that luzindole treatment recovered the cytokine production in CLP mice, which was downregulated by melatonin treatment. Interestingly, the peritoneal macrophages in naive mice could not phagocytose *E. coli*, which was consistent with the results of a previous study (48). Moreover, phagocytic activities of peritoneal macrophages against *E. coli* and *S. aureus* were not affected by melatonin treatment. Thus, these data suggest that melatonin may control the cytokine production in macrophages of CLP mice, but the melatonin-treated macrophages may not contribute to the clearance of the invading bacteria during polymicrobial infection in mice. To define the effect of MT1 and MT2 in the neutrophils and macrophages, neutrophil- and macrophage-specific MT1 and MT2 knockout mice should be investigated for the melatonin-induced anti-septic effects.

The bactericidal effects of neutrophils are mediated by phagocytosis, degranulation, ROS release, and NETs (10, 49). Although the phagocytic activity of neutrophils is the well-studied form of their anti-bacterial effect, recent studies have shown that NETs produced by neutrophils also effectively kill bacteria (13, 42). In this study, we found that melatonin treatment did not enhance, but rather decreased, the phagocytic activities of neutrophils, whereas it promoted NET formation. Both phagocytosis and NETs contribute to the clearance of bacteria, while NETs inhibit the phagocytosis of bacteria in neutrophils (13, 42). The induction of NETs by melatonin treatment also clearly defined that the bacteria trapping into NETs, may promote killing of the bacteria. Moreover, the inhibition of MT2 by luzindole prevented NET formation in response to melatonin treatment and bacterial infection, consequently resulting in the failure to inhibit the colonization of bacteria in polymicrobial-infected mice. Therefore, these data suggested that melatonin promoted the contribution of NETs in the clearance of bacterial pathogens in polymicrobial-infected mice.

Treatment with melatonin in CLP mice induced NET formation, whereas the naive neutrophils did not develop NETs upon melatonin treatment. *S. aureus* infection has been shown to induce NET formation (50). Therefore, the melatonin treatment may enhance the development of NETs during bacterial infections possibly by autophagy and superoxide production (51). Autophagy is another defense mechanism against polymicrobial infection (52, 53). Therefore, the induction of NETs by melatonin may also be able to promote autophagy in neutrophils. In contrast with this, superoxide is inhibited by melatonin during sepsis (54), even though superoxide is one of the important factors for induction of autophagy (51). Therefore, future studies are required to elucidate the role of NETs, autophagy, and superoxide in the inhibition of sepsis in mice.

In conclusion, in this study, we found that melatonin promoted NET formation during polymicrobial infection, which had an anti-bacterial effect. Combined with the anti-inflammatory effect of melatonin, the anti-bacterial effect of melatonin-induced NETs could be potentially useful for human patients with sepsis.

## Ethics Statement

All the experiments were carried out under the guidelines of the Institutional Animal Care and Use committee at the SPHCC. The mouse method (2018-A050-01) was approved by the committee on the Ethics of Animal Experiments of the SPHCC.

## Author Contributions

J-OJ designed the experiments and wrote the manuscript. LX, WZ, MK, and LZ participated in the experiments and data analysis. PL and MK reviewed the article. PL helped with manuscript writing, and made important corrections to the manuscript. The final version of manuscript was approved by all authors.

### Conflict of Interest Statement

The authors declare that the research was conducted in the absence of any commercial or financial relationships that could be construed as a potential conflict of interest.
