# Supplementary material for: Protective Effect of Melatonin Against Polymicrobial Sepsis Is Mediated by the Anti-bacterial Effect of Neutrophils
Source: Front Immunol. 2019 Jun 19;10:1371. doi: 10.3389/fimmu.2019.01371 (PMC6593141; doi:10.3389/fimmu.2019.01371)
Supplement: Supplementary file 1 [file Data_Sheet_1.pdf]

Supplementary Material

1 Materials and methods

1.1 Analysis of AST levels

The AST analysis kit was purchased from Sigma-Aldrich, and AST levels were measured according to the standard laboratory techniques.[1]

1.2 Analysis of apoptosis

Mouse spleens were harvested, and single-cell suspensions were prepared as described previously.[2,3] Splenocytes were stained with Annexin V-FITC and 7AAD in 100 μL of a binding buffer for 15 min at room temperature (RT). After the addition of 400 μL of binding buffer, cells were analyzed by flow cytometry using a FACS aria II (Becton Dickinson).

1.3 TUNEL assay

Paraffin-embedded tissue sections were deparaffinized, hydrated, and then subjected to an *in situ* apoptosis assay, using Trevigen TACS XL *In Situ* Apoptosis detection kit (R&D Systems) in accordance with the manufacturer's instructions.

1.4 ELISA

The concentrations of IL-6, IL-17, IL-10, IFN-γ, and TNF-α in the peritoneal fluid were measured in triplicate, using standard ELISA kits (BioLegend, San Diego, CA, USA).

1.5 PCR primer

The primer sequences are as follows: mouse β-actin forward, 5′-TGGATGACGATATCGCTGCG-3′, reverse, 5′-AGGGTCAGGATACCTCTCTT-3′, MT1 forward, 5′-TATCCACACTGGTTGGGGTC-3′, reverse, 5′-GTTCAGGTTTGGTCTGTTGCT-3′, and MT2 forward, 5′-TTCATCCAGACAGCCAGCAC-3′, reverse, 5′-TTCCTTGACAGGCACGGTAG-3′.

2 Supplementary results

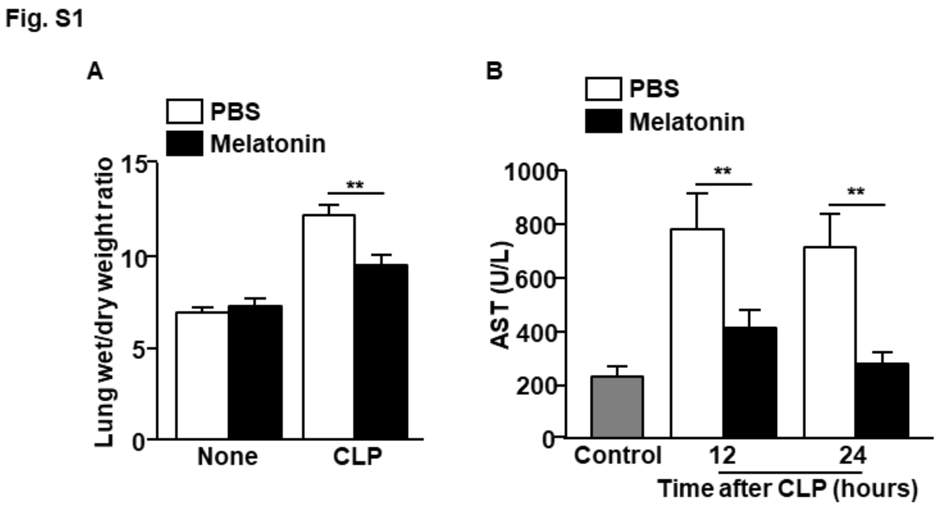

Supplementary Figure 1. Melatonin reduced the CLP-induced peripheral tissue damage in mice. Mice were treated as indicated in Fig. 1 and then subjected to CLP surgery. (A) The lung wet/dry weight ratio 24 h after CLP surgery is shown. (B) Levels of plasma AST were measured 12 h and 24 h after CLP surgery. Data are the average of six independent measurements (two mice per experiment, for a total of three experiments). *$p < 0.05$, **$p < 0.01$.

Fig. S2

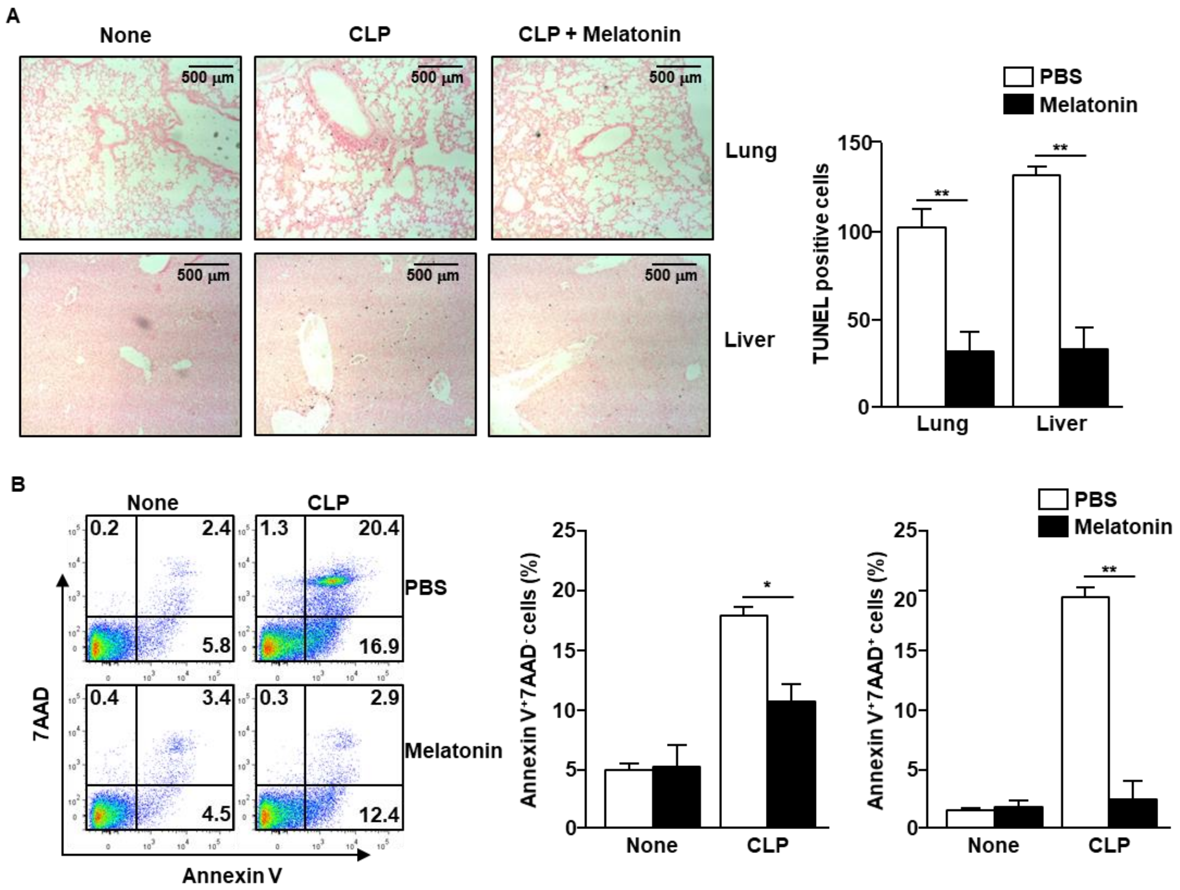

Supplementary Figure 2. Melatonin inhibited the cecal ligation puncture (CLP)-induced apoptosis of peripheral tissue cells in mice. Mice were injected with 1 mg/kg melatonin for 30 min and then subjected to CLP surgery. After the surgery, the mice further received melatonin at the same concentration. (A) The lung and liver tissues were subjected to TUNEL assay 24 h after CLP surgery (left panel). Black dots indicate the dead cells. The mean number of TUNEL-positive cells in lung and liver (right panel). (B) Apoptosis of splenocytes was determined by Annexin V/7AAD staining 24 h after the CLP surgery (left panel). The mean percentages of early apoptosis (Annexin $V^{+}7AAD^{-}$; middle panel) and late apoptosis (Annexin $V^{+}7AAD^{+}$; right panel). All data represent the average of six independent measurements (two mice per experiment, for a total of three experiments). *$p < 0.05$, **$p < 0.01$.

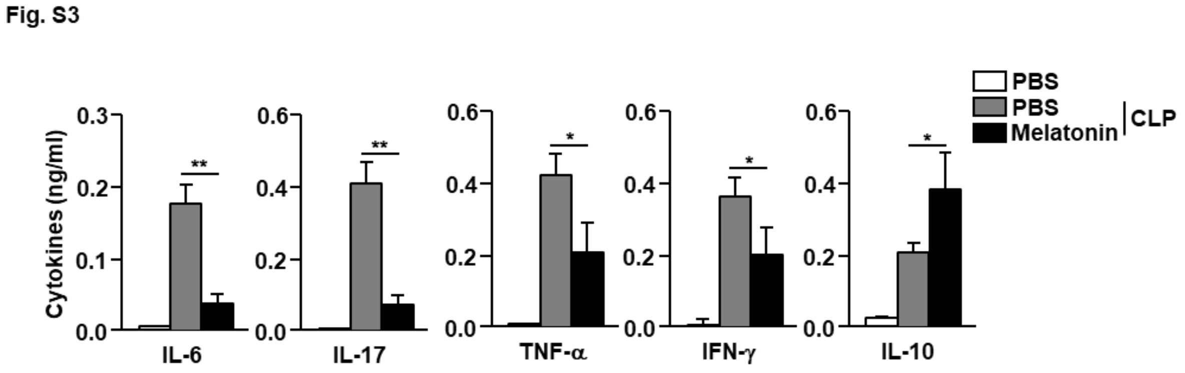

Supplementary Figure 3. Melatonin reduced the inflammatory cytokine production in CLP mice. Mice were treated with 1 mg/kg melatonin, as shown in Fig. 1. Twenty-four hours after the CLP surgery, sera were harvested and the concentrations of the indicated cytokines in the sera were measured by ELISA. Data are the average of six independent measurements. *$p < 0.05$, **$p < 0.01$.

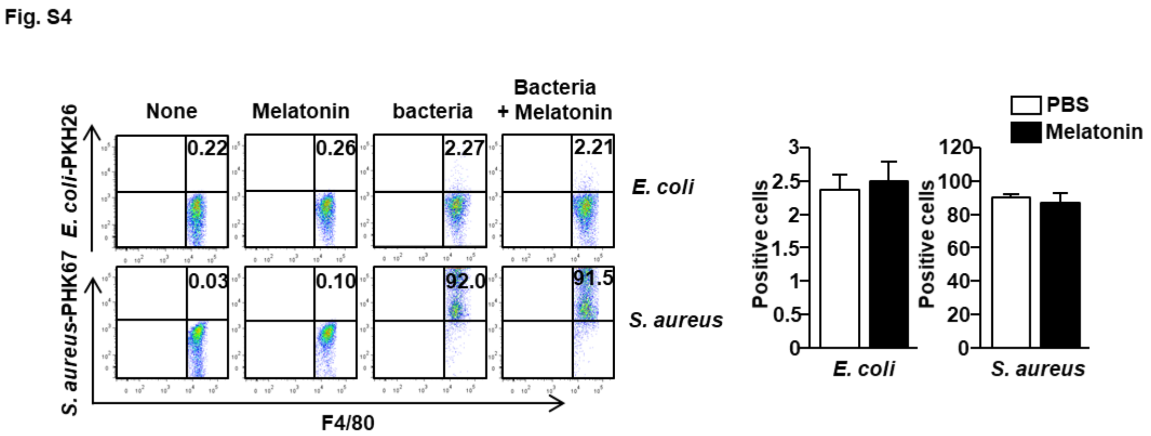

Supplementary Figure 4. Melatonin did not enhance the phagocytic activity of macrophages against *E. coli* and *S. aureus*. Isolated macrophages from the peritoneum were treated with melatonin and co-cultured with PKH26-labeled *E. coli* and PKH67-labeled *S. aureus* for 1 h. The phagocytosis of bacteria was measured by flow cytometry after staining with F4/80 (Left panel). The mean levels of *E. coli* phagocytosis (middle panel) and *S. aureus* (right panel) are shown. Data represent the average of six independent measurements.

Fig. S5

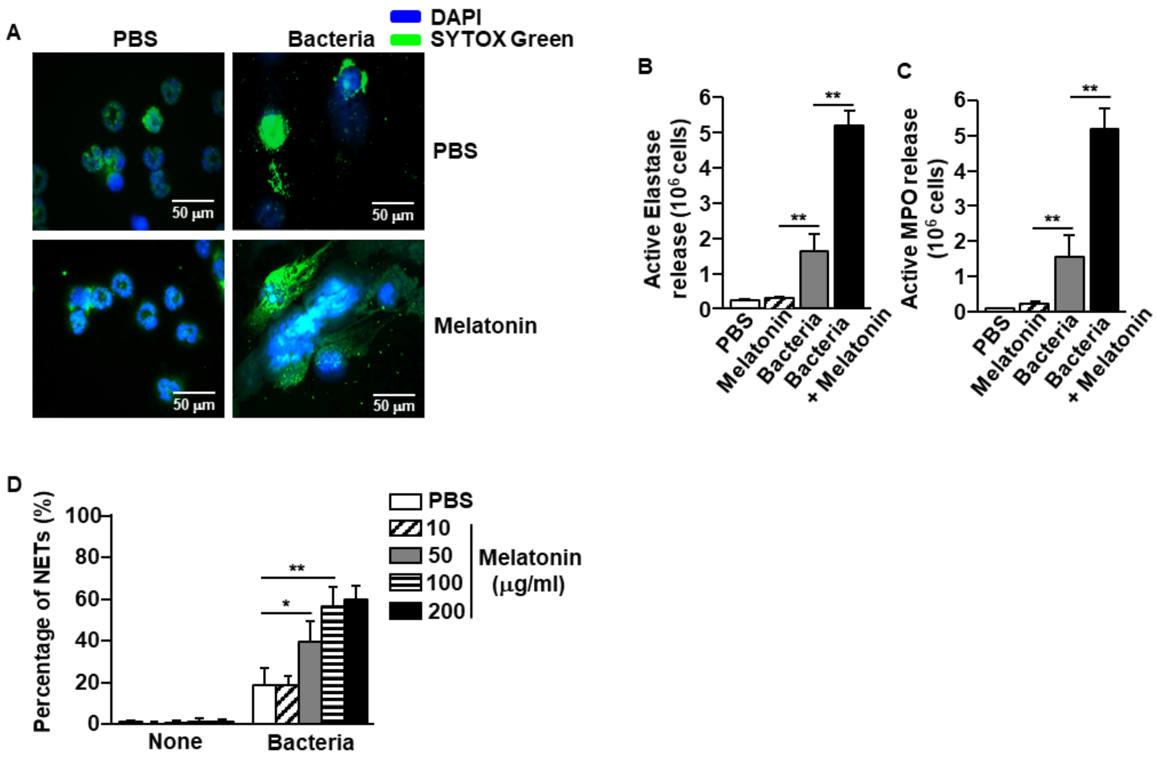

Supplementary Figure 5. Melatonin induced neutrophil extracellular traps (NETs) during bacterial infection. Isolated neutrophils from bone marrow were co-cultured with a mixture of *E. coli* and *S. aureus* for 6 h, in the presence and absence of melatonin (100 μg/mL). (A) The neutrophils were spun down on a glass slide and stained with SYTOX Green and DAPI. (B) Active elastase release levels in the cultured medium were measured after treatment of 100 μg/mL of melatonin and bacteria. (C) Active MPO release levels are shown. (D) The isolated neutrophils were treated with indicated dose of melatonin and co-cultured with a mixture of *E. coli* and *S. aureus* for 6 h. The percentage of NETs was measured after spinning down the cells on the glass slide. All data are from six individual samples within each group (three mice per experiment, with a total of two independent experiments). *$p < 0.05$, **$p < 0.01$.

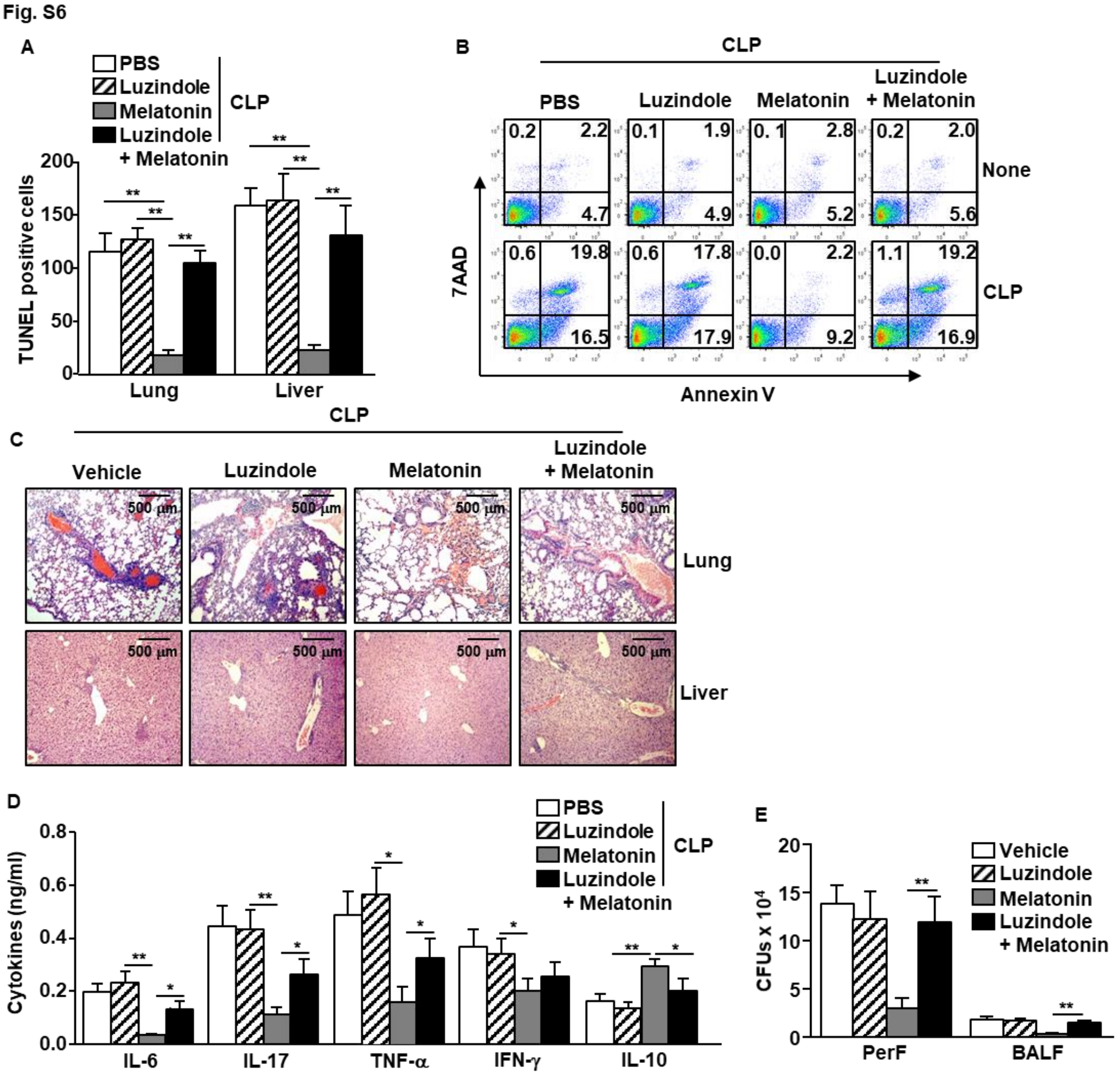

Supplementary Figure 6. Luzindole inhibited the melatonin-induced protective effect against polymicrobial infection in mice. C57BL/6 mice were pretreated with 5 mg/kg of luzindole for 1 h and then with 1 mg/kg of melatonin. Thirty minutes after melatonin treatment, the mice underwent CLP surgery and were injected with the same concentration of luzindole and melatonin again. (A) Mean number of TUNEL-positive cells in the lung and liver, analyzed 24 h after the CLP surgery. (B) Apoptosis of the splenocytes was measured by Annexin V/7AAD staining. Data are from six individual mice within each group. ***$p < 0.01$*. (C) H&E staining of lung and liver sections, 24 h after the CLP surgery. (D) Serum concentrations of the indicated cytokines were measured by ELISA. Data represent the average of six independent samples. (E) CFUs in PerF and BALF were measured 24 h after CLP surgery. Data are from six individual mice within each group. **$p < 0.05$, *$p < 0.01$*.

Fig. S7

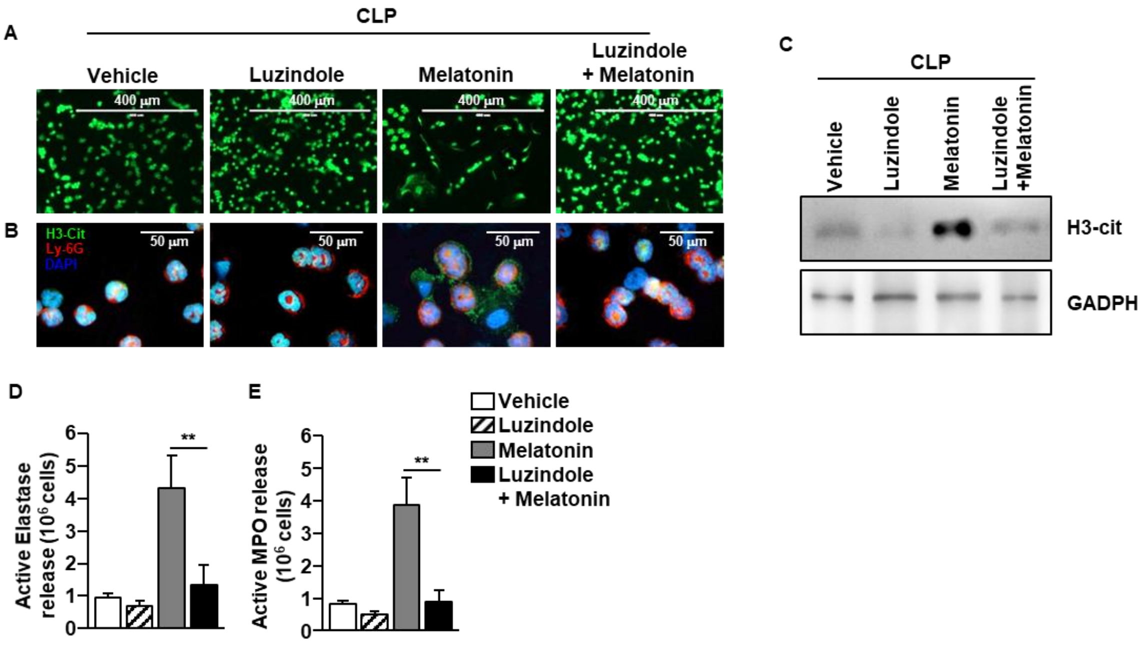

Supplementary Figure 7. Luzindole countered the melatonin-induced inhibition NET formation. Mice were treated with luzindole and melatonin and subjected to CLP surgery, as indicated in Fig. S6. (A) Peritoneal cells were stained with SYTOX Green after 6 h of CLP surgery. (B) The peritoneal cells were analyzed using immunofluorescence staining with Ly-6G, H3-cit, and DAPI. (C) Secreted protein levels of H3-cit were measured in the peritoneal fluid. (D) The active elastase and (E) myeloperoxidase (MPO) levels were measured in the peritoneal fluid 6 h after CLP surgery. All data represent the average of six independent samples (two mice per experiment, for a total of three experiments). ***p* < 0.01.

References

1. Horder M, Gerhardt W, Harkonen M, et al. Experiences with the Scandinavian recommended methods for determinations of enzymes in blood. A report by the Scandinavian Committee on Enzymes (SCE). *Scandinavian journal of clinical and laboratory investigation.* 1981;41(2):107-116.

2. Jin JO, Han X, Yu Q. Interleukin-6 induces the generation of IL-10-producing Tr1 cells and suppresses autoimmune tissue inflammation. *Journal of autoimmuni ty.* 2013;40:28-44.

3. Jin JO, Zhang W, Du JY, Wong KW, Oda T, Yu Q. Fucoidan can function as an adjuvant in vivo to enhance dendritic cell maturation and function and promote antigen-specific T cell immune responses. *PloS one.* 2014;9(6):e99396.
